# Terms of the specialized nursing language in the care of older adults at home

**DOI:** 10.1590/1980-220X-REEUSP-2022-0138en

**Published:** 2023-03-31

**Authors:** Bianca Bueno Paz, Bruna Karen Cavalcante Fernandes, Jorge Wilker Bezerra Clares, Jardeliny Corrêa da Penha, Maria Augusta Rocha Bezerra, Angelina Monteiro Furtado

**Affiliations:** 1 Universidade Federal do Piauí, Floriano, PI, Brazil.; 2 Universidade Estadual do Ceará, Fortaleza, CE, Brazil.

**Keywords:** Nursing, Standardized Nursing Terminology, Aged, Home Health Nursing, Home Care Services, Enfermagem, Terminologia Padronizada em Enfermagem, Idoso, Enfermagem Domiciliar, Serviços de Assistência Domiciliar, Enfermagem, Terminologia Padronizada em Enfermagem, Idoso, Enfermagem Domiciliar, Serviços de Assistência Domiciliar

## Abstract

**Objective:**

To identify terms of the specialized nursing language used in the care of older adults at home and map them with the International Classification for Nursing Practice.

**Method:**

This is a methodological study, operationalized by the steps of extraction of terms from the specialized nursing language in the care of older adults at home from official documents; normalization; cross mapping between extracted terms and those included in the International Classification for Nursing Practice, 2019/2020 version; distribution according to the Seven-Axis Model.

**Results:**

A total of 12,365 terms were identified and, after manual screening, 530 terms were included, which were mapped with the International Classification for Nursing Practice and analyzed according to the level of equivalence, resulting in the presence of 460 (86.8%) terms, 375 (70.7%) with level of equivalence 1 and 85 (16.0%) with level of equivalence 2; and 70 (13.2%) non-included terms, 34 (6.4%) with level of equivalence 3, 22 (4.1%) with level of equivalence 4 and 14 (2.6%) with level of equivalence 5.

**Conclusion:**

The terms identified will serve as a basis for the elaboration of diagnoses, results, and nursing interventions for older adults living at home.

## RESUMO

**Objective:**

Identificar termos da linguagem especializada de enfermagem utilizados no cuidado à pessoa idosa domiciliada e mapeá-los com a Classificação Internacional para a Prática de Enfermagem.

**Método:**

Estudo metodológico, operacionalizado pelas etapas: extração de termos da linguagem especializada de enfermagem no cuidado à pessoa idosa domiciliada a partir de documentos oficiais; normalização; mapeamento cruzado entre termos extraídos e os constantes na a Classificação Internacional para a Prática de Enfermagem, versão 2019/2020; distribuição segundo Modelo de Sete Eixos.

**Resultados:**

Identificaram-se 12.365 termos e após a triagem manual foram incluídos 530 termos, que foram mapeados com a Classificação Internacional para a Prática de Enfermagem e analisados de acordo com o grau de equivalência, resultando em 460 (86,8%) termos constantes, sendo 375 (70,7%) com grau de equivalência 1 e 85 (16,0%) com grau de equivalência 2; e 70 (13,2%) termos não constantes, sendo 34 (6,4%) com grau de equivalência 3, 22 (4,1%) com grau de equivalência 4 e 14 (2,6%) com grau de equivalência 5.

**Conclusão:**

Os termos identificados servirão de base para a elaboração de diagnósticos, resultados e intervençōes de enfermagem para pessoas idosas domiciliadas.

## RESUMEN

**Objetivo:**

Identificar términos del lenguaje especializado de enfermería utilizados en el cuidado de ancianos en el domicilio y mapearlos con la Clasificación Internacional de la Práctica de Enfermería.

**Método:**

Estudio metodológico, operacionalizado por las etapas: extracción de términos del lenguaje especializado de enfermería en el cuidado de ancianos que viven en sus casas a partir de documentos oficiales; normalización; mapeo cruzado entre los términos extraídos y los contenidos en la Clasificación Internacional de la Práctica de Enfermería, versión 2019/2020; distribución según el modelo de siete ejes.

**Resultados:**

Se identificaron 12.365 términos y luego de la clasificación manual se incluyeron 530 términos, los cuales fueron mapeados con la Clasificación Internacional de la Práctica de Enfermería y analizados según el grado de equivalencia, resultando 460 (86,8%) términos constantes, 375 (70,7%) con grado de equivalencia 1 y 85 (16,0%) con grado de equivalencia 2; y 70 (13,2%) términos no constantes, 34 (6,4%) con grado de equivalencia 3, 22 (4,1%) con grado de equivalencia 4 y 14 (2,6%) con grado de equivalencia 5.

**Conclusión:**

Los términos identificados servirán como base para la elaboración de diagnósticos, resultados e intervenciones de enfermería para personas mayores que viven en el hogar.

## INTRODUCTION

Due to changes arising from the aging process, older adults require specific care, which represents a major challenge for the Brazilian health system. In this context, home care has become an important strategy to expand this population’s access to health services, especially for dependent people, those with disabilities and/or in vulnerable situations, whose purpose is to ensure the continuity of care at home and reduce hospitalizations^(1)^. Its organization takes place in different modalities, with the first level being represented by the Primary Health Care (PHC) teams^(2)^.

The nurse is part of the minimum composition of the multidisciplinary home care team, playing a central role in the development of actions aimed at maintaining, improving or recovering health, enhancing the maximum possible physical and psychological well-being and independence in activities of daily living^(1)^. For the effectiveness of their clinical practice, these professionals have to use scientific knowledge and standardized language to support the nursing process, resulting in the provision of systematic and quality care, focused on the older adult’s needs at home^(3)^.

In this context, the use of classification systems is of great importance, with emphasis on the International Classification for Nursing Practice (ICNP^®^), whose structure of terms and definitions allows systematically collecting, describing and documenting the elements of professional nursing practice. This terminology is an integral part of the global information infrastructure on health care practices and policies worldwide, representing the domain of Nursing in the World Health Organization (WHO) Family of International Classifications^(4)^, and has recently been incorporated into SNOMED CT, the world’s most comprehensive clinical terminology^(5)^.

The use of ICNP^®^ allows the identification, validation, and mapping of useful terms and concepts for clinical nursing practice, which can be used in the elaboration of diagnoses, results, and nursing interventions aimed at structuring terminological subsets for specific populations and priorities, contributing to the improvement of the terminology^(6)^.

Specialized nursing terminologies were evidenced in the literature for the care of older adults in different contexts, namely community elderly^(7)^, elderly women with HIV/AIDS^(8)^ and for the prevention of falls in the elderly in PHC^(9)^, with a scarcity of studies related to older adults living at home. The construction of a specialized nursing terminology for this clientele envisages contributing to the advancement of nurses’ clinical practice in PHC, as its use can improve communication among professionals, records and clinical reasoning in view of the needs of this population, providing more safety and quality to the care offered and collaborating with professional autonomy in decision-making^(10)^.

In view of the above, the present study aimed to identify terms of the specialized nursing language used in the care of older adults at home and map them with the ICNP^®^.

## METHOD

### Design of Study

This is a methodological study, conducted in two stages, following the Brazilian guidelines for the development of specialized nursing terminologies based on the ICNP^®(11)^: 1) identification of relevant terms for professional nursing practice with older adults living at home; 2) cross-mapping of identified terms with ICNP ^®^ 2019/2020 version terms.

### Data Analysis and Treatment

The study was carried out from February to June 2021.

In the first stage, two official documents published by the Brazilian Ministry of Health were analyzed^(12,13)^ for the identification of terms considered useful for professional nursing practice with older adults living at home. These documents were chosen because they are reference guides for family health teams in the health care of older adults and for home care.

The documents were compiled into a single file in the software *Word for Windows* ^®^, excluding graphic accents and sections with low potential to contain relevant terms (credits section, author identification, table of contents, objectives, and references). Eventually, this document was converted to the *Portable Document Format* (PDF) to allow the extraction of terms through the software PORONTO^(14)^, which transformed the *corpus* into a list of simple and compound terms, which was exported to an *Excel for Windows* ^®^ spreadsheet.

Subsequently, the listed terms underwent an analysis by the main author and were revised by two other study authors, independently, to exclude repetitions and linking elements, terms related to medical procedures, diseases and medications and that were not considered useful for clinical practice with the elected priority. Disagreements were discussed among researchers to reach a consensus. It should be noted that these researchers have a graduate degree (one doctor and two masters) and academic and professional experience of more than five years in the health area for the elderly and in the use of ICNP®.

The terms were manually normalized in terms of spelling, gender, number and degree, standardized with terms of ICNP^®^ 2019/2020 version, and arranged in alphabetical order. Adjectives and nouns were normalized preferably in the masculine gender and singular, and verbs were normalized in the infinitive (in Portuguese).

In the second stage, the normalized terms were cross-mapped with the terms of the ICNP Seven-Axis Model^®^ 2019/2020 version, through the software *Access for Windows* ^®^, resulting in a spreadsheet containing terms included and not included in this classification.

**Chart 1:**
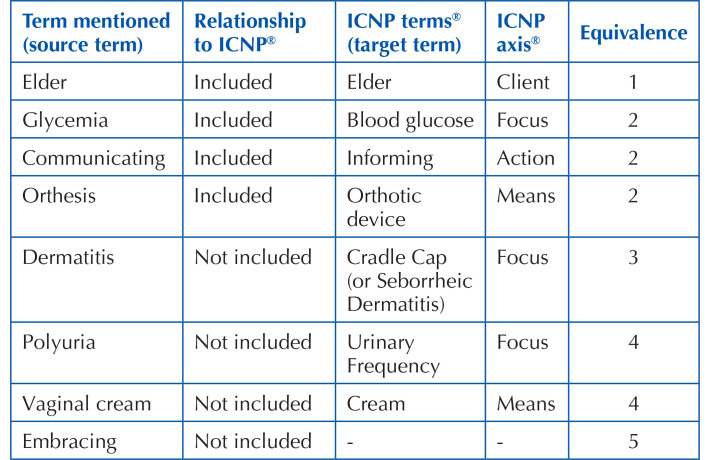
Examples of terms mapped in the study, with analysis of the level of equivalence between source terms and target terms – Floriano, PI, Brazil, 2021.

Both the terms included and non-included in ICNP^®^ were analyzed regarding the mapping level of equivalence according to the recommendations of the ISO/TR 12300:2016 Standard, being classified into: 1 – equivalence of lexical and conceptual meaning; 2 – equivalence of meaning, but with synonymy; 3 – source term is broader and has less specific meaning than the target term; 4 – source term is more restricted and has more specific meaning than the target term; 5 – no mapping is possible ^(15)^, as exemplified in Chart 1 below.

After this process, the terms evaluated with equivalence 1 and 2 were grouped in the set of included terms identified in the mapping, being replaced by the ICNP equivalent terms^®^ with their respective classification codes. The terms evaluated with level of equivalence 3, 4 or 5 were grouped in the set of non-included terms, and were classified according to the Seven Axis Model: Action, Client, Focus, Judgment, Location, Means, and Time.

### Ethical Aspects

This study was not submitted for approval by the Research Ethics Committee, since only the literature was used as a data source, without involving human beings.

## RESULTS

In the first stage, the automatic extraction of 12,365 terms was carried out. Of these, 530 terms were considered useful for professional nursing practice with older adults living at home.

In the second stage, the 530 selected terms were mapped with the terms included in the ICNP^®^ 2019/2020 version, of which 460 (86.8%) were identified as included terms, 375 (70.7%) with level of equivalence 1 and 85 (16.0%) with level of equivalence 2; and 70 (13.2%) non-included terms, with 34 (6.4%) being of level of equivalence 3, 22 (4.1%) with level of equivalence 4, and 14 (2.6%) with level of equivalence 5, as shown in Table 1.

**Table 1.** Distribution of terms identified in the study, according to ICNP axes® and levels of equivalence – Floriano, PI, Brazil, 2021.

AXES	Level 1 n(%)	Level 2 n(%)	Level 3 n(%)	Level 4 n(%)	Level 5 n(%)	Total n(%)
Focus	229 (43.2)	25 (4.7)	27 (5.1)	17 (3.2)	4 (0.7)	302 (57.0)
Judgment	17 (3.2)	4 (0.7)	–	–	–	21 (4.0)
Means	45 (8.5)	4 (0.7)	6 (1.1)	4 (0.7)	6 (1.1)	65 (12.3)
Action	30 (5.7)	46 (8.7)	–	–	2 (0.4)	78 (14.7)
Time	7 (1.3)	2 (0.4)	–	–	–	9 (1.7)
Location	41 (7.7)	4 (0.7)	1 (0.2)	1 (0.2)	1 (0.2)	48 (9.0)
Client	6 (1.1)	–	–	–	1 (0.2)	7 (1.3)
**Total**	**375 (70.7)**	**85 (16.0)**	**34 (6.4)**	**22 (4.1)**	**14 (2.6)**	**530 (100)**

**Chart 2:**
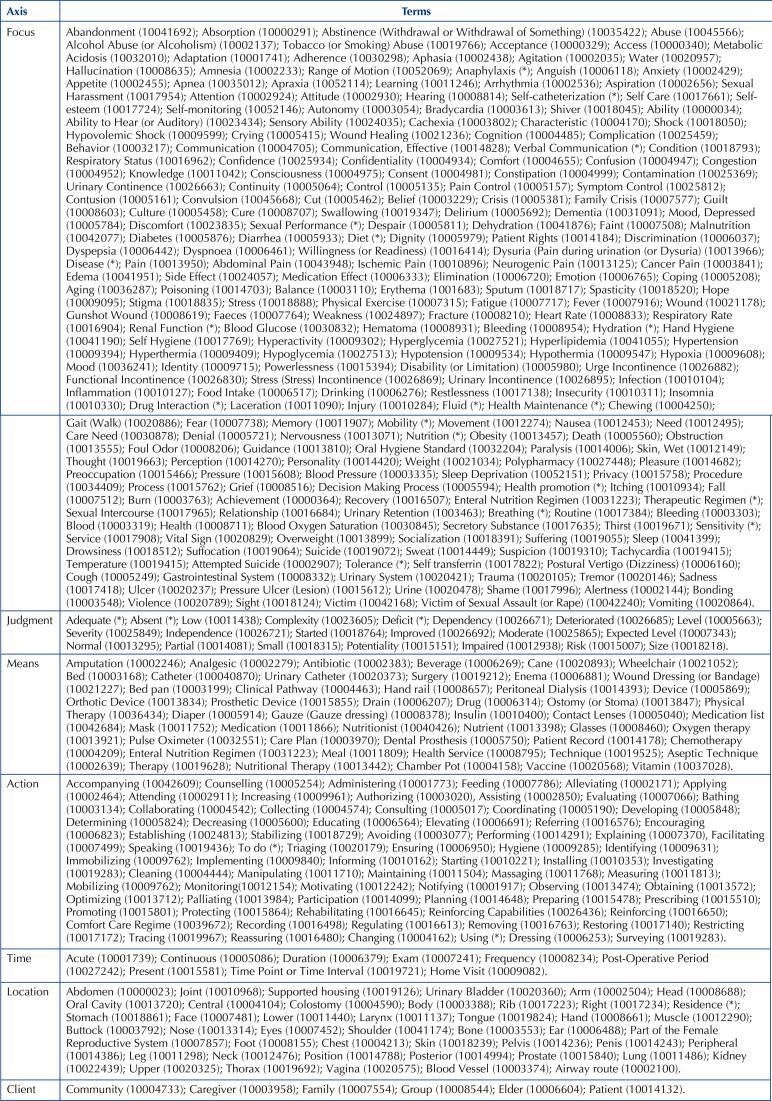
Terms identified in the study and included in the ICNP® Version 2019/2020 – Floriano, PI, Brazil, 2021.

**Chart 3:**
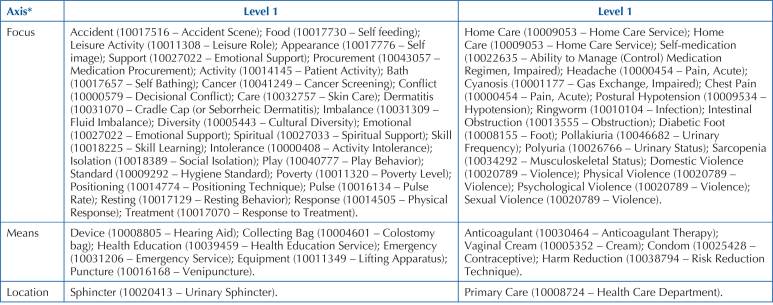
Terms identified in the study (source terms), classified with levels of equivalence 3 and 4 and with their respective ICNP® target terms – Floriano, PI, Brazil, 2021.

**Chart 4:**
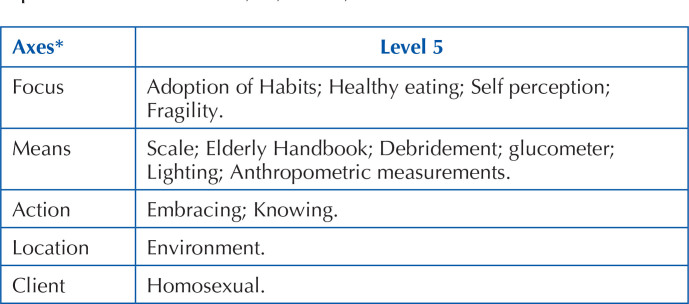
Terms identified in the study and classified with level of equivalence 5 – Floriano, PI, Brazil, 2021.

In Charts 2, 3 and 4, the terms identified in the study can be seen, distributed according to the Seven Axis Model.

*Are present at ICNP®, but they do not exist as atomic terms, so they have no code.

*No terms were identified in the Judgment, Action, Time and Customer axes.

*No terms were identified in the Judgment and Time axes.

## DISCUSSION

In the present study, the number of terms classified as included in the ICNP^®^ 2019/2020 version was significant. This finding corroborates other studies that developed specialized terminology using the ICNP^®(7,8)^. This demonstrates that ICNP^®^ is a terminology that has been contemplating many terms of the nurse’s clinical practice with older adults.

On the other hand, the non-included terms identified in this study point to the possibility of including these new terms in this classification system, with great potential to represent the clinical practice phenomena in the care of older adults at home.

The Focus axis grouped the largest number of terms, which can be explained by the fact that this axis is related to the relevant area of care for nursing. Similar results were evidenced in other terminological studies^(16,17)^.

Among the terms identified in the study and classified in the Focus axis, there are terms related, for the most part, to aspects commonly observed in nursing practice in the care of older adults at home, such as: “Dehydration”, “Malnutrition”, “Constipation”, “Diarrhea”, “Urinary Incontinence”, “Urinary Tract Infection”, “Pain”, “Fall”, “Burn”, “Hypertension”, “Hypotension” and “Polydrug (or Polypharmacy)”. These terms reflect biological and physiological aspects of care, since the aging process influences the emergence of comorbidities, leading to increased vulnerability, which can lead to reduced functionality and contribute to the sickening process. In this scenario, the nurse shall develop a systematized care plan centered on the needs of the older adults at home, aiming at improving their health, empowerment, independence and safety ^(18)^.

With regard to the psychological aspects of care, still in the Focus axis, we can highlight the included terms: “Abandonment”, “Anguish”, “Anxiety”, “Self-esteem”, “Autonomy”, “Mood, Depressed”, “Memory”, “Preocupation”, “Suicide” and “Sadness”. These terms encompass concepts of the mental process that explore the affective or emotional complexities that can compromise the elder’s quality of life and well-being. Nurses’ participation in the monitoring of this population should consider the investigation of signs of mental suffering, aiming at the prevention and early detection of psychological symptoms and the promotion of mental health, to ensure healthy aging ^(19)^.

In this context, the terms “Socialization” and “Communication” stand out, classified in the Focus axis, which cover the context of the elder’s interactive behavior at home and in the community, being an important factor for active participation in society, as well as for building and keeping social relationships with a view to promoting the elder’s autonomy. Thus, the nurse can intervene to promote and stimulate the participation of this population in social activities, which can be carried out in groups.

The terms “Belief”, “Culture” and “Hope”, classified in the Focus axis, cover the aspects of spirituality, which occupies a prominent place in the elder’s life, being recognized as an important internal resource that helps older adults to face the difficulties and stressful events, especially the health-disease process. Thus, the importance of nurses in understanding the spiritual dimension for providing nursing care that integrates the body, mind and spirit, from health promotion to rehabilitation, is highlighted ^(20)^.

Regarding the Means axis, the included terms related to technological, therapeutic and technical resources that cover concepts that help in the care of older adults at home were identified, such as; “Cane”, “Wheelchair”, “Bed”, “Wound Coverage (or Bandage)”, “Diaper”, “Dental Prosthesis”, “Vaccine”, “Analgesic”, “Antibiotic”, “Ostomy”, “Insulin”, “Medication” and “Aseptic Technique”. Such terms are already part of the concepts of nursing interventions included in the ICNP^®^_;_ therefore, they have the potential to improve the description of nurses’ actions with a view to ensuring comprehensive care according to these individuals’ needs.

The terms classified in the Action axis are related to the construction of nursing information necessary to guide the older adult, the family and/or caregiver in the management of care, such as: “Applying”, “Relieving”, “Assisting”, “Evaluating”, “Sanitizing (or Hygiene Care)”, “Mobilizing”, “Paliating”, “Reassuring” and “Dressing”; in the recovery and rehabilitation of the elder’s health, such as “Administering”, “Feeding”, “Encouraging”, “Maintaining”, “Motivating”, “Prescribing”, “Protecting”, “Rehabilitating”, “Reinforcing” and “Restoring” ; also focused on educational practices, such as “Accompanying”, “Advising”, “Educating”, “Explaining”, “Facilitating” and “Promoting”. We emphasize the importance of these terms for the elaboration of nursing interventions aimed at assisting in the planning of nursing care for the older adult at home, providing better quality of life and promoting his/her autonomy.

It is also worth highlighting the included term “Restricting (or restraining)”, classified in the Action axis, which reflects the manual method to limit the elder’s mobility, with the purpose of controlling agitation, avoiding possible falls, and preventing the removal of the health devices ^(21)^. In the literature, there are controversies about the safety, efficacy and efficiency of using this method in the older adult at home, due to the negative repercussions related to the increase in undesirable damage. In view of this, the need for the nursing team and elder’s caregivers’ knowledge, guidance and monitoring is reinforced to prevent the occurrence of injuries in this clientele ^(21)^.

Also in this axis, attention is drawn to the term Comfort Care Regime (or Palliative Care), classified with equivalence 2 (Palliative Care), since this is significant for the care of the older adult at home. In this care scenario, the nurse’s performance allows a differentiated look to meet the demands presented by these users, developing care actions that promote comfort, pain relief, quality of life and protection of human dignity, equipping caregivers and family members for the home palliative care ^(13)^.

With regard to the non-included terms allocated in the Means axis, the following were identified: terms related to devices contributing to the care plan of the older adult at home (“Elder’s Handbook”); to nutritional care assistance (“Scale”, “Glucometer”, “Anthropometric measurements”); to the prevention of falls (“Lighting”); to the treatment and prevention of sexually transmitted infections (“Vaginal Cream”, “Condom”); to care related to the circulatory system (“Puncture”, “Anticoagulant”) and tegumentary system (“Debridement”). The terms of this axis express methods of how to carry out nursing interventions to this clientele, as well as the relevance of updating new terms in the ICNP^®^.

Among the non-included terms, the term “Embracing” stands out, classified in the Action axis, which expresses nursing actions guided by humanized care. The act of embracing fosters qualified listening, ensures the bond and the resolution of care, based on the uniqueness of the elder to meet their needs, being indispensable in the elder’s nursing ^(22)^.

The term “Homosexual”, classified in the Client axis, is not included in the ICNP^®^. This finding is relevant, since homosexuality in old age is still little addressed, especially in health services, which requires public policies aimed at accessing and assisting this public, who often do not assume their sexual orientation, for fear of prejudice, social stigma, and lack of confidence^(23)^. Thus, it is up to health professionals, especially nurses, to be trained to provide care that allows identifying and understanding the specificities of this clientele, as well as expanding assistance for adherence to the Brazilian Public Health System and provide these people’s biopsychosocial well-being.

It should also be noted that some terms classified in this study as included, namely “Anaphylaxis”, “Self-catheterization”, “Verbal Communication”, “Sexual Performance”, “Diet”, “Disease”, “Kidney Function”, “Hydration”, “Drug Interaction”; “Liquid”, “Health Maintenance”, “Mobility”, “Nutrition”, “Health Promotion”, “Therapeutic Regimen”, “Breathing”, “Sensitivity” and “Tolerance”, classified in the Focus axis; “Adequate”, “Absent” and “Deficit”, classified in the Judgment axis; “Do” and “Use”, classified in the Action axis and “Home”, classified in the Location axis, are present in ICNP^®^, but not as atomic terms classified on an axis, but as part of the concepts of Diagnosis/Outcome and/or Nursing Intervention.

This finding revealed an inconsistency in the ICNP^®^ hierarchy and made it difficult to analyze and classify some terms. To overcome this gap, the review of some ICNP^®^ atomic terms present in the concepts is suggested, aiming at classifying them in one of the seven axes, thus facilitating the use of combinatorial terminology in care practice and in research on the construction of specialized terminology for priority areas and groups.

As a limitation of the study, the search for relevant terms in only two official documents stands out, which may restrict the scope of the results. Despite this, it is believed that the presented terminology has the potential to become a significant mediator for the clinical practice of nursing with older adults at home, since it can help the nurse in the use of a standardized language, as well as provide autonomy to perform the care planning in a systematic way, guided by clinical judgment, also facilitating interprofessional communication and records in the field of action.

## CONCLUSION

In compliance with the objective of this study, 530 terms relevant to nursing care for elderly people at home were identified, 460 of which are included and 70 are not included in the ICNP^®^ 2019/2020 version.

In case this study is continued, these terms will be used in the construction of statements of diagnoses, results and nursing interventions, which will serve as a basis for structuring an ICNP^®^ terminological subset aimed at this clientele.
